# Evaluating the impact of a newly established hospitalist medicine procedure service on length of stay and procedural outcomes: A retrospective propensity‐matched analysis

**DOI:** 10.1002/jhm.70311

**Published:** 2026-03-29

**Authors:** Jeremy Gentile, Saria Alazmeh, Kriti Soni, Tarek Alsibai, Felicia Wright, Alicia Stowe, Fergus Peacock

**Affiliations:** ^1^ Corewell Health Grand Rapids Michigan USA; ^2^ Government Medical College Chandigarh India; ^3^ Stormont Vail Topeka Kansas USA

## Abstract

**Background:**

Reliance on interventional radiology (IR) has been associated with delays in procedure completion and prolonged hospital length of stay (LOS). Hospitalist medicine procedure services (HMPS) have been proposed as a strategy to mitigate these inefficiencies, though outcomes of newly established programs composed of physicians with minimal ultrasound training remain underexplored.

**Objective:**

To evaluate the clinical and operational impact of a newly implemented HMPS composed of hospitalists with minimal prior procedural experience.

**Methods:**

We conducted a retrospective propensity‐matched cohort study at two urban hospitals in Grand Rapids, Michigan, including 920 admitted patients undergoing paracentesis or thoracentesis between April 2021 and May 2023. Patients were matched 1:1 between those treated by IR before HMPS implementation and those treated by HMPS postimplementation. Primary outcomes were LOS and time from procedure order to completion. Secondary outcomes included serious complications.

**Results:**

Following matching (*N* = 460 per group), HMPS was associated with a 14.8% reduction in LOS (*p* = 0.001) and a significant reduction in median time from order to completion compared with IR (794.5 vs. 2049.5 min, *p* < 0.001). Complication rates were low and not significantly different between groups. Multivariable modeling identified order‐to‐completion time and antithrombotic use as independent predictors of LOS.

**Conclusions:**

A newly created hospitalist procedure service, staffed by clinicians with limited procedural experience, significantly reduced LOS and procedural delays without compromising safety. Findings suggest that expanding procedural capacity and reducing time‐to‐procedure is an effective strategy to improve efficiency and throughput in hospitals where IR‐based workflows create bottlenecks.

## INTRODUCTION

In 2006, the American Board of Internal Medicine (ABIM) removed procedural volume requirements for thoracentesis and paracentesis from internal medicine training standards.^1^ While the exact reasoning is not well documented, procedural literature cites declining performance rates,^2^ inconsistent supervision,^3^ low comfort level with ABIM′s required procedural training volumes,^4^ and time constraints related to duty hour limits^5^ as key contributing factors. Multiple studies have subsequently reported persistently low levels of procedural competence and confidence among internal medicine physicians.^2^, ^3^, ^4^

Increasing reliance on specialized providers, particularly interventional radiology (IR), for these procedures has resulted in increased time to procedural completion compared with bedside performance. Emerging evidence supports the use of hospitalist‐run hospital medicine procedure services (HMPS) as an effective strategy to mitigate these delays, with studies demonstrating reductions in length of stay (LOS) and time to procedure without increased complication rates or diminished diagnostic yield.^6^, ^7^, ^8^, ^9^, ^10^, ^11^, ^12^, ^13^, ^14^, ^15^, ^16^ Additionally, bedside procedures rather than radiology referral have been associated with increased patient satisfaction^17^ and increased resident participation and competency.^7^

Prior studies have largely focused on established procedure teams at large academic centers. Health systems seeking to establish internal procedure services may hesitate due to concerns about training efficacy, procedural safety, and whether newly launched teams can replicate the benefits observed in mature programs. This study describes the creation of a newly implemented HMPS with limited or no prior ultrasound‐guided procedural experience and evaluates the impact of this service on patient outcomes and hospital operations. We compare LOS, time from order to procedure completion, and complication rates between procedures performed by the HMPS after implementation and procedures performed by IR preimplementation across a multihospital system in Michigan.

## METHODS

### HMPS structure

The HMPS was created in October 2022 and is composed of board‐certified, practicing, adult hospitalist physicians who began with minimal or no procedural experience. Participating physicians were trained to perform ultrasound‐guided paracentesis and thoracentesis. The primary training site, Corewell Health Butterworth Hospital, is a large, high‐acuity tertiary referral center serving western Michigan. The second site, Corewell Health Blodgett Hospital, is a large community hospital within the same integrated health system.

### Hospitalist training

Training began in late October 2022, when two experienced procedural hospitalists with existing procedural credentialing were reassigned from regional hospital sites to the main campus at Corewell Health Butterworth Hospital to serve as trainers. This hospitalist‐led training model required planned backfilling of regional clinical responsibilities, which was incorporated into the operational design of the training period. In total, 13 hospitalists were trained over 16 weeks through this structured approach. Because a credentialed HMPS provider staffed all procedures beginning on Day 1 of the pilot, no washout period existed in which both the HMPS and IR performed comparable procedural volumes concurrently.

Following completion of the initial training phase, the HMPS transitioned to a sustainable operational model that supports both procedural throughput and ongoing clinical care 7 days a week. Each hospital site is staffed daily by a single credentialed proceduralist who carries approximately half of a standard inpatient census while performing procedures. Further information about the structure and creation of the procedure service can be found in Appendix SA.

### Patient selection

After successful implementation of the HPMS, we conducted a retrospective cohort study across Corewell Health Butterworth and Blodgett Hospitals in Grand Rapids, MI, from April 1, 2021, to May 1, 2023. Hospitalized (observation or admission) adult patients ( ≥ 18 years) who underwent thoracentesis or paracentesis performed by IR or the HMPS were eligible for inclusion. Procedures were identified using a structured electronic medical record (EMR) query of consult orders (hospitalist procedure vs. IR procedure) and were verified by review of procedure notes. Patients who underwent a procedure while in the emergency department but were discharged before admission were excluded from the analysis.

### Demographics

Data on age, gender, race, ethnicity, comorbid conditions, complications, and hospital admission date/time and discharge date/time were obtained from the EMR. Comorbidity was assessed using the diagnosis‐related group (DRG) weight as a surrogate for patient complexity. Anticoagulation and antiplatelet exposure were captured both before hospital admission and during hospitalization.

### Outcomes

Primary outcomes were actual hospital LOS and time from procedure order to completion. For IR, completion time was determined using the time from consult order placement to the procedure start flag in the IR procedure log, which is entered by nursing staff when the patient is brought into the suite and the procedure is initiated. For HMPS procedures, the order‐to‐completion interval was defined as the time from consult order placement to the timestamp of the finalized procedure note in the EMR. This approach was used because the bedside HMPS did not utilize a dedicated nursing flowsheet or procedure start flag, requiring reliance on provider documentation to determine completion time.

Secondary outcomes included procedural complications: pneumothorax, major bleeding (defined as the need for red blood cell transfusion within 24 h after the procedure), transfusion of fresh frozen plasma (FFP) or platelets, intensive care unit (ICU) transfer within 24 h after the procedure, and mortality both at 48 h (to capture immediate morality potentially related to the procedure) and during the hospital admission. Cases completed by newly trained HMPS attendings were included in the overall procedure volume, ensuring that any adverse events related to procedural training were represented in the study cohort.

After implementation, the HMPS assumed responsibility for the majority of inpatient paracentesis and thoracentesis procedures. To minimize bias, the matched patients were divided into two cohorts: those treated by IR pre‐HMPS implementation and those treated by the HMPS post‐HMPS implementation. This approach avoided confounding introduced by the postimplementation referral pathway in which patients with complex anatomy, prior adverse events, or sedation needs were preferentially triaged to IR.

### Statistical analysis

Propensity‐score matching was performed using 1:1 greedy matching to balance the HMPS and IR cohorts. Greedy matching without replacement was selected to ensure a stable 1:1 matched cohort and prevent overrepresentation of individual cases. We controlled for variables including age, sex, race, comorbidities (e.g., cirrhosis, heart failure, diabetes), DRG weight, anticoagulation before hospital admission (direct oral anticoagulants or warfarin), and procedure type. Antithrombotic medication use during hospitalization (anticoagulation, aspirin, clopidogrel, or ticagrelor) was not included in the propensity score matching because it was prespecified as a covariate of interest to be evaluated independently in multivariable regression modeling. Patients who underwent multiple admissions or multiple procedures were eligible for inclusion; however, only the first qualifying procedure per patient was included in the matched cohort. The Charlson Comorbidity Index (CCI) was calculated after propensity score matching and was not included as a matching covariate.

Statistical analysis included Wilcoxon Rank Sum tests for nonparametric comparisons and multivariable linear regression with backward selection to identify independent predictors of LOS. A Bonferroni–Holm correction was applied to adjust for multiple comparisons.

We performed multivariable log‐linear regression with backward elimination to determine predictors of LOS. Candidate variables included proceduralist service, procedure type, time from order to procedure completion, and antithrombotic use during hospitalization. Because baseline covariates were balanced through propensity score matching, postmatching regression was intentionally restricted to prespecified explanatory variables rather than reintroducing matching covariates, to avoid overadjustment. A log transformation was applied to account for the non‐normal distribution.

## RESULTS

### Hospital medicine proceduralists

Baseline procedural exposure varied considerably among HMPS physicians: 30.8% (*n* = 4) reported no prior ultrasound experience of any kind, 15.4% (*n* = 2) reported limited medical school exposure without subsequent experience, 38.5% (*n* = 5) had ultrasound training or procedural experience during residency but none as attendings, and 15.4% (*n* = 2) had completed a professional training course or ultrasound fellowship as attendings. On average, HMPS physicians completed 6.6 paracentesis procedures (SD 4.2, range 13) and 11.2 thoracentesis procedures (SD 6.4, range 20) before procedural comfort was achieved.

### Demographics

A total of 920 patients were included in the matched cohort (460 per group). Patient demographics, comorbidities, and medication use were not significant between groups after propensity score matching (Table 1). Post hoc comparison demonstrated similar mean CCI values between the HMPS and IR groups, suggesting no major imbalance in comorbidity burden. A descriptive table of unmatched patients is provided in Table 1, Appendix SB, and demonstrates broadly similar characteristics to the matched cohort. Antithrombotic medication use during hospitalization was similar between groups (Table 2, Appendix SB).

**Table 1:** Patient characteristics before and after propensity score matching.

	Before propensity score matching			After propensity score matching		
Variable	Hospitalist, *N* = 500	IR, *N* = 1800	*p* Value	Hospitalist, *N* = 460	IR, *N* = 460	*p* Value
Age, years (mean ± SD)	66.8 ± 15.6	65.2 ± 15.6	0.04	67.3 ± 15.3	67.2 ± 14	0.90
Race						
White or Caucasian	422 (84.4%)	1558 (86.6%)	0.22	387 (84.1%)	388 (84.3%)	0.93
Black or African American	40 (8.0%)	91 (5.1%)	0.01	38 (8.3%)	42 (9.1%)	0.64
Asian	6 (1.2%)	15 (0.8%)	0.45	6 (1.3%)	3 (0.7%)	0.51
Other	32 (6.4%)	136 (7.6%)	0.38	29 (6.3%)	27 (5.9%)	0.78
Male sex	245 (49.0%)	1070 (59.4%)	<0.001	230 (50%)	229 (49.8%)	0.95
Comorbidities						
Heart failure	269 (53.8%)	878 (48.8%)	0.05	251 (54.6%)	232 (50.4%)	0.21
Cirrhosis	188 (37.6%)	488 (27.1%)	<0.001	172 (37.4%)	176 (38.3%)	0.79
Chronic kidney disease	191 (38.2%)	613 (34.1%)	0.09	180 (39.1%)	173 (37.6%)	0.64
Diabetes	218 (43.6%)	690 (38.3%)	0.03	202 (43.9%)	209 (45.4%)	0.64
Hypertension	409 (81.8%)	1367 (75.9%)	0.006	380 (82.6%)	378 (82.2%)	0.86
Aspirin prior to admission	9 (1.8%)	90 (5.0%)	0.002	9 (2.0%)	7 (1.5%)	0.61
Anticoagulation prior to admission	140 (28.0%)	333 (18.5%)	<0.001	132 (28.7%)	129 (28.0%)	0.83
Clopidogrel/ticagrelor prior to admission	33 (6.6%)	67 (3.7%)	0.005	32 (7.0%)	34 (7.4%)	0.80
Platelet count prior to procedure (median [IQR])	*N *= 499 202 [126, 292]	*N* = 1786 206 [134, 316]	0.07	*N* = 459 200 [125, 289]	*N* = 457 191 [123, 306]	0.87
INR > 2 prior to procedure	20 (4.5%)	30 (1.7%)	<0.001	18 (4.4%)	11 (2.4%)	0.10
DRG weight, median (IQR)	*N* = 460 1.58 [1.20, 1.95]	*N* = 1786 1.8 [1.30, 2.40]	<0.001	1.6 [1.20, 1.96]	1.7 [1.20, 1.90]	0.49
Charlson Comorbidity Index (mean ± SD)	9.5 ± 4.7	9.8 ± 4.5	0.21	9.4 ± 4.7	9.8 ± 4.4	0.23
Procedure type						
Paracentesis	239 (47.8%)	681 (37.8%)	<0.001	223 (48.5%)	231 (50.2%)	0.60
Thoracentesis	261 (52.2%)	1119 (62.2%)	<0.001	237 (51.5%)	229 (49.8%)	0.60

Abbreviations: INR, international normalized ratio; IQR, interquartile range; IR, interventional radiology.

**Table 2:** Hospital metrics.

Outcome	Hospitalist (*N* = 460)	Interventional radiology (*N* = 460)	Adjusted *p* Value
Length of stay, days (median [IQR])	7 [4, 12]	8 [5, 14.5]	0.03
Length of stay by antithrombotic use during admission, days (median [IQR])			
Yes	8 [5, 15]	10 [6, 19]	0.18
No	7 [4, 12]	7 [4, 13]	0.45
Length of stay by procedure type, days (median [IQR])			
Paracentesis	8 [4, 13]	8 [4, 14]	1.00
Thoracentesis	7 [4, 12]	9 [6, 15]	0.01
Billed DRG geometric mean length of stay, days (median [IQR])	4.3 [3.5, 5]	4.6 [3.6, 4.9]	1.00
Time from order to completion of procedure, minutes (median [IQR])	794.5 [283, 1342.5]	2049.5 [1265, 3132.5]	0.002
Admission time to paracentesis and thoracentesis, minutes (median [IQR])	2608 [1376.5, 6854.5]	4741 [2667.5, 9258]	0.002

Abbreviation: IQR, interquartile range.

### Primary outcomes

Median hospital LOS was significantly shorter in the HMPS group compared with the IR group (7 vs. 8 days; *p* = 0.002). Median time from order‐to‐completion was 794.5 min (13.25 h) for HMPS and 2049.5 min (34.14 h) for IR (*p* < 0.001), translating to a reduction of 1255 min (20.92 h) per procedure. Admission‐to‐procedure time was similarly reduced (2608 vs. 4741 min; *p* < 0.001) (Table 2).

Procedures performed on weekends accounted for 28% of HMPS and 25.4% of IR cases, with no significant differences in order‐to‐completion time or LOS between weekend and weekday procedures (Table 3, Appendix SB).

**Table 3:** Clinical outcomes.

Outcome	Hospitalist (*N* = 460)	Interventional radiology (*N* = 460)	Adjusted *p* Value
Mortality within 48 h of procedure	3 (0.7%)	8 (1.7%)	1.00
In‐hospital mortality	48 (10.4%)	46 (10.0%)	1.00
Pneumothorax	5 (1.1%)	0 (0.0%)	0.66
Transfer to ICU within 24 h	9 (2.0%)	4 (0.9%)	1.00
RBC transfusion within 24 h	1 (0.2%)	0 (0.0%)	1.00
FFP or platelet transfusion within 24 h	12 (2.6%)	13 (2.8%)	1.00

Abbreviations: ICU, intensive care unit; RBC, red blood cell.

### Secondary outcomes

Complication rates were low and similar between groups. Pneumothorax occurred in 1.1% of HMPS patients versus 0% in IR and was not statistically significant. The need for blood products, ICU transfers within 24 h, and both 48‐h and in‐hospital mortality were not significantly different between groups (Table 3).

### Regression analysis

Although procedural service type was significantly associated with a 14.8% reduction in LOS in unadjusted analyses (*p *= <0.001), this association was no longer statistically significant after adjustment for order‐to‐completion time (*p* = 0.1162). Additionally, while thoracentesis initially demonstrated a statistically significant difference in LOS for group comparisons, univariate modeling (with LOS as the dependent variable) failed to confirm procedure type as a significant independent predictor (*p* = 0.1164). After elimination of procedure group and procedure type, the final regression model predicting LOS was built using antithrombotic medication use and time from order to completion as independent variables.

Based on multivariable regression analysis, the impact of antithrombotic use during hospitalization amounted to a 33.5% increase in LOS. Each additional hour from order to procedure completion was independently associated with a 0.36% increase in LOS. Estimated mean LOS ranged from 6.79 days for HMPS patients not receiving antithrombotic therapy during their hospital stay to 9.78 days for IR patients receiving antithrombotic therapy during their hospital stay (Table 4).

**Table 4:** Predicted length of stay based on service‐specific median timing.

Antithrombotic medication status	Hospitalist timing LOS in days [95% CI]	Interventional radiology timing LOS in days [95% CI]
No antithrombotic medications	6.79 [6.38, 7.23]	7.33 [6.90, 7.78]
On antithrombotic medications	9.07 [8.23, 9.98]	9.78 [8.94, 10.70]

Abbreviations: CI, confidence interval; LOS, length of stay.

## DISCUSSION

Earlier investigations, such as those by *Ritter* et al. and *Nanan* et al., largely described mature HMPS programs within academic centers, often staffed by faculty with preexisting procedural competency or dedicated certification. To our knowledge, this is the first study to expressly evaluate the clinical outcomes of a newly created HMPS composed of physicians with minimal prior procedural experience. Despite nearly half of the trainees (46.2%) having no procedural exposure since medical school and 84.7% never having performed these procedures autonomously, the HMPS achieved a 1255‐min reduction in order‐to‐completion time and a 1‐day reduction in median LOS without compromising patient safety.

The 12.5% relative improvement in LOS represents a substantial operational gain that may translate to significant financial benefit for hospitals, particularly those with high procedure volumes or prolonged wait times at baseline. Crucially, our findings highlight a scalable staffing structure where proceduralists maintain clinical continuity by carrying a partial patient census (0.5 full‐time equivalent). This integration of procedural and clinical duties (a detail about specific team structure often omitted from earlier studies) suggests that the benefit of a hospitalist‐led service is not contingent on a specialized “proceduralist‐only” role. This application may be particularly relevant for community hospitals, where procedural demand may be too low to justify a “procedure‐only” hospitalist, but still exceeds available subspecialty capacity. In this situation, training existing hospitalists to perform procedures and round on hospitalized patients may be the most scalable and cost‐effective solution.

An important consideration in interpreting the difference in time to procedure completion is the structural difference in how procedural timing was recorded between services. IR timestamps reflected procedure initiation, whereas HMPS timestamps reflected completion of the procedure as well as documentation. Consequently, any observed reduction in order‐to‐completion time for the HMPS is likely an underestimate of the true operational difference between the two services.

Weekend procedure rates did not differ meaningfully between cohorts, and there were no significant differences in LOS or procedure timeliness between weekday and weekend cases. This finding contrasts with prior literature suggesting that expanded weekend coverage drives LOS reduction^13^ and supports the interpretation that the primary mechanism of benefit was reduction in general procedural bottlenecks rather than expansion of weekend capacity alone. At our institution, IR continued to provide weekend coverage throughout the study period, which may have attenuated any incremental benefit of HMPS availability on weekends. In settings where IR does not routinely staff weekends, the impact of a 7‐day‐a‐week hospitalist‐led procedure service on LOS might be even more pronounced.

Notably, both anticoagulant use and procedural timing remained significant in the final regression model, while the group performing the procedure lost statistical significance in multivariate analysis. These findings suggest that while the group performing the procedure seemed to be the initial driver of improved LOS, the more proximate determinant is rather the time delay between ordering and performing the procedure. This is further supported by the regression analysis suggesting a 0.36% increase in LOS for every hour between order placement and procedural completion. Over the time intervals typically observed in practice, this effect accumulates substantially; for example, a 24‐h delay corresponds to an estimated 8.6% increase in LOS. It is notable that the nearly 21‐h reduction in procedure turnaround time closely paralleled the 1‐day reduction in LOS, supporting a plausible causal mechanism linking procedural efficiency with earlier discharge.

This efficiency, however, is not merely a matter of team speed but can be deeply influenced by patient‐level factors and system‐specific protocols, specifically anticoagulant washout requirements. In multivariable analysis, antithrombotic medication use during the hospital stay emerged as a major independent predictor, increasing LOS by 33.5%. This supports the interpretation that system‐level antithrombotic workflows, including institutional washout practices around aspirin, P2Y12 inhibitors, and oral anticoagulants, meaningfully influence throughput independent of procedural ownership. Because the IR service employed a stricter anticoagulation washout policy than recommended in the Society of Interventional Radiology guidelines^18^ utilized by the HMPS, these clinical “holds” may have acted as a practical bottleneck in the IR cohort.

A key strength of this study is the integration of propensity score matching with multivariable regression analysis, a rigorous dual‐methodological approach. While propensity score matching successfully balanced baseline clinical characteristics including age, comorbidities, and procedural indications to create comparable cohorts, the subsequent multivariable regression was essential for identifying the specific drivers of LOS within those balanced groups. This tiered analysis allowed us to distinguish between the “identity” of the performing service and the “efficiency” of the procedural workflow. By confirming that the LOS benefit was mediated by order‐to‐completion time rather than the service itself, our approach provides a level of internal validity and causal insight that simpler observational designs often lack.

Ultimately, these data indicate that the primary institutional benefit of a hospitalist‐led procedure service lies in the optimization of procedural timeliness and the resolution of workflow bottlenecks, rather than the specific clinical setting or the identity of the performing team.

Although the study was not powered to detect rare safety events, the absolute complication rates were low in both cohorts and consistent with published norms,^19^ reinforcing the safety of bedside procedural care performed by newly trained hospitalists. As noted previously, training‐phase procedures were incorporated into the analytic cohort to ensure early‐phase safety signals were captured; however, supervised versus independent procedures could not be separately analyzed due to inconsistent coding in the EMR. Of note, all HMPS pneumothoraces were reviewed, with 80% representing ex vacuo changes or trapped lungs rather than iatrogenic injury. Similarly, the patient who required a blood transfusion in the HMPS cohort had no signs of hemothorax or bleeding, and their anemia was secondary to chronic malignancy. This contextualization is important, as raw complication counts likely overstate clinically meaningful risk.

This study has several limitations. As a retrospective cohort study, residual confounding is possible, and temporal changes unrelated to service implementation may have contributed to the observed LOS differences. A time–interaction term was not included in the final model due to limited statistical power and the short interval of observation; however, the short study window and close alignment between reductions in procedural timeliness and LOS mitigate this concern. Minor complications, such as postprocedural leaks, postparacentesis acute kidney injury, minor bleeding, or transient hypotension, were not included due to limited documentation. Additionally, we did not evaluate long‐term mortality, as given the very small complication rate and overall long lengths of hospital stay, we did not feel this would commonly be attributable to the procedure itself.

Overall, these results reinforce that the value of an HMPS is not merely in shifting procedural ownership but in redesigning care delivery pathways to minimize bottlenecks that extend hospitalization. Results of this study are likely to have the largest degree of impact on those facilities with prolonged order‐to‐completion times or substantial procedural backlogs, particularly outside of academic medical centers.

## CONCLUSION

Our study captures outcomes during the formative phase of a community‐based hospitalist‐led procedure service, directly addressing institutional concerns about feasibility, training efficacy, and early safety. Despite being staffed by physicians with limited prior procedural experience, we report no statistically significant difference in serious complication rates when compared with IR.

By employing propensity‐score matching and multivariable regression, we isolated the operational drivers of HMPS efficiency, revealing that the observed reduction in LOS was primarily mediated by a substantial decrease in time from order to procedure completion rather than the specific procedural service. These findings underscore the critical importance of addressing procedural bottlenecks, including system‐driven medication washout requirements, when designing inpatient workflows. Together, these features position our study as the first to rigorously quantify the early impact of a newly established HMPS in a nonuniversity setting, providing both methodological and translational insights for institutions considering similar programs.

Future research should evaluate the financial and clinical implications of procedure timing across a broader range of diagnostic and therapeutic interventions, as well as explore strategies to optimize workflow, training, and resource utilization for bedside procedural programs.

## CONFLICT OF INTEREST STATEMENT

The authors declare no conflicts of interest.

## ETHICS STATEMENT

This study was approved by the Corewell Health IRB.

## Supporting information

Appendix A.

Appendix B.
