# Supplementary material for: Evaluating the impact of a newly established hospitalist medicine procedure service on length of stay and procedural outcomes: A retrospective propensity‐matched analysis
Source: J Hosp Med. 2026 Mar 29;21(10):1134–41. doi: 10.1002/jhm.70311 (PMC13636170; doi:10.1002/jhm.70311)
Supplement: Supplementary file 1 [file JHM-21-1134-s001.docx]

**Appendix A:**

*HMPS Details and Structure*

The HMPS was created in October 2022 after internal audits demonstrated a steady increase in radiology procedural backlogs, particularly when requests were categorized as “noncritical,” and during holidays and weekends. It was hypothesized that implementation of a dedicated HMPS could reduce turnaround time by more than 50%, with a corresponding decrease in hospital length of stay and associated cost savings.

Initial annual procedural volume prior to creation of the HMPS was approximately 1,800 thoracenteses and paracenteses yearly. The HMPS now performs approximately 80% of all inpatient paracenteses and thoracenteses across two urban hospitals in Grand Rapids.

Funding for the development of the HMPS was approved by the Division of Hospital Medicine following an internal financial analysis demonstrating an anticipated positive return on investment (ROI). This calculation balanced projected reductions in hospital length of stay and increased IR throughput of higher-value procedures against the relatively low cost of training. Stakeholders involved in HMPS development included hospitalist division leadership, IR, interventional pulmonology, nursing leadership, laboratory leadership, and operations staff supporting the Division of Hospital medicine and informatics. Clinical pathways related to anticoagulation management and the structured evaluation of ascites and pleural effusion were developed by physician leads in hospital medicine, pulmonology, and gastroenterology and approved by system committees. Ultrasound equipment was procured separately as part of a system-wide expansion of POCUS but was incorporated into HMPS workflows. In addition, a dedicated EMR procedure order set, modeled after existing IR workflows, was created and linked to a consult tracking list, with patients automatically removed upon completion of the procedural note.

Prior to program initiation, only 1 of the performing hospitalists (excluding trainers) held existing procedural credentialing with most physicians having prior ultrasound exposure confined to residency or medical school. During the 16-week training period, participating hospitalists had their clinical census reduced by four to six patients to accommodate one to four procedures per day during their seven-day service block. This reduction in patient volume was absorbed by the remaining hospitalist team without additional staffing. The trainers’ regional hospital shifts were temporarily backfilled at cost, requiring coverage for 112 hospitalist shifts during the training phase and representing a modest, short-term investment that enabled rapid establishment of internal procedural expertise while maintaining continuity of care across the health system.

Designated procedural trainers maintained approximately half of a standard team census (6–8 patients versus 12–16) during the training period, to allow sufficient time for procedures and bedside instruction while maintaining direct patient care responsibilities. Continuous training and procedural coverage were intentionally structured by alternating the trainers into seven-day service blocks for the duration of the program.

Each trainee completed a minimum of 5 successful supervised paracenteses and thoracenteses under the direct observation of the credentialed trainer before being approved for hospital privileging with additional procedures performed as needed to achieve procedural comfort. Once all 13 physicians were fully trained and credentialed, the service was expanded to include the smaller community hospital, Corewell Health Blodgett Hospital, where nearly all paracenteses and thoracenteses subsequently transitioned to the HMPS.

After transition to the final model, at Corewell Health Butterworth Hospital, higher procedural volumes necessitated the addition of an internal medicine resident to assist with immediate clinical care and facilitate daily workflow, while also providing ongoing educational exposure to bedside procedures. In contrast, at Corewell Health Blodgett Hospital, procedural volumes are lower and the proceduralist operates independently. This structure allows proceduralists to maintain clinical continuity while preserving capacity to perform bedside procedures throughout the day. Operationally, this model represents a dedicated increased cost (over strict patient rounding) of approximately one proceduralist per day (0.5 FTE at each hospital), equivalent to two full-time equivalents (FTE) annually (assuming 182 shifts per hospitalist). In addition to the capture of procedural wRVUs, ongoing hospitalist staffing costs are balanced against system-level benefits, including reduced length of stay, improved interventional radiology throughput, and a shift toward a higher relative-value case mix for IR. The service therefore functions not only as a procedural capacity solution but also as a targeted operational investment intended to improve hospital efficiency and resource utilization.
