# Supplementary material for: Evaluating the impact of a newly established hospitalist medicine procedure service on length of stay and procedural outcomes: A retrospective propensity‐matched analysis
Source: J Hosp Med. 2026 Mar 29;21(10):1134–41. doi: 10.1002/jhm.70311 (PMC13636170; doi:10.1002/jhm.70311)
Supplement: Supplementary file 2 [file JHM-21-1134-s002.docx]

**Appendix B:**

**Table 1. Patients Not Included in Propensity Score Matching**

| **Variable** | **Hospitalist**  **N = 40** | **IR**  **N = 1340** |
| --- | --- | --- |
| Age | 60.9 ± 17.5 | 64.5 ± 16.0 |
| Use of Aspirin Prior To Admission | 0 (0%) | 83 (6.2%) |
| Race |  |  |
| White or Caucasian | 35 (87.5%) | 1170 (87.3%) |
| Black or African American | 2 (5.0%) | 49 (3.7%) |
| Asian | 0 (0%) | 12 (0.9%) |
| Other | 3 (7.5%) | 109 (8.1%) |
| Sex (male) | 15 (37.5%) | 841 (62.8%) |
| Comorbidities |  |  |
| Heart Failure | 18 (45.0%) | 646 (48.2%) |
| Cirrhosis | 16 (40.0%) | 312 (23.3%) |
| Chronic Kidney Disease | 11 (27.5%) | 440 (32.8%) |
| Diabetes | 16 (40.0%) | 481 (35.9%) |
| Hypertension | 29 (72.5%) | 989 (73.8%) |
| Anticoagulation prior to admission | 8 (20.0%) | 204 (15.2%) |
| Use of clopidogrel / ticagrelor prior to admission | 1 (2.5%) | 33 (2.5%) |
| Last platelet count prior to procedure (median [IQR]) | N = 40  226 [152.5, 335] | N = 1329  213 [139, 318] |
| INR > 2 PTA | 2 (5.7%) | 19 (1.4%) |
| Charlson Comorbidity Index (mean ± SD) | 10.5 ± 4.8 | 9.8 ± 4.6 |
| Procedure Type |  |  |
| Paracentesis | 16 (40.0%) | 450 (33.6%) |
| Thoracentesis | 24 (60.0%) | 890 (66.4%) |

**Table 2. Antithrombotic Use During Hospital Stay**

| **Variable** | **Hospitalist (N = 460)** | **Interventional Radiology**  **(N = 460)** | **Adjusted p-value** |
| --- | --- | --- | --- |
| Anticoagulation during hospital stay | 56 (12.2%) | 49 (10.7%) | 1.0 |
| Aspirin during hospital stay | 93 (20.2%) | 101 (22.0%) | 1.0 |
| Clopidogrel/ ticagrelor during hospital stay | 11 (2.4%) | 14 (3.0%) | 1.0 |
| Any antithrombotic (anticoagulant or antiplatelet) during hospital stay | 134 (29.1%) | 139 (30.2%) | 1.0 |

**Table 3. Time to Procedure Completion and Length of Stay by Weekday vs Weekend**

|  | **Hospitalist** | | | **Interventional Radiology** | | |
| --- | --- | --- | --- | --- | --- | --- |
| **Outcome** | **Weekday**  **(N = 331)** | **Weekend**  **(N = 129)** | **p-value** | **Weekday**  **(N = 343)** | **Weekend**  **(N = 117)** | **p-value** |
| Time to from order to completion of procedure, minutes (median [IQR]) | 777 [281, 1266] | 878 [285, 1430] | 0.32 | 1936 [1258, 3144] | 2153 [1360, 2924] | 0.56 |
| Length of stay, days (median [IQR]) | 7 [4, 12] | 8 [4, 14] | 0.84 | 9 [5, 14] | 8 [5, 15] | 0.52 |

**Table 4. Multivariable Regression Model for Actual Length of Stay**

| **Predictor Variable** | **95% CI** | **% Change in LOS** | **p-value** |
| --- | --- | --- | --- |
| Intercept (Baseline) | (1.79, 1.94) | - | <0.001 |
| Antithrombotic medication use | (0.18, 0.40) | +33.5% | <0.001 |
| Time from order to completion (per 60 min) | (0.0017, 0.0055) | +0.36% | <0.001 |
